# Eye-Head Coordination Abnormalities in Schizophrenia

**DOI:** 10.1371/journal.pone.0074845

**Published:** 2013-09-10

**Authors:** Simon Schwab, Othmar Würmle, Nadja Razavi, René M. Müri, Andreas Altorfer

**Affiliations:** 1 Department of Psychiatric Neurophysiology, University Hospital of Psychiatry and University of Bern, Bern, Switzerland; 2 Center for Cognition, Learning and Memory, University of Bern, Bern, Switzerland; 3 Perception and Eye Movement Laboratory, Departments of Neurology and Clinical Research, Inselspital and University of Bern, Bern, Switzerland; CNRS - Université Claude Bernard Lyon 1, France

## Abstract

**Background:**

Eye-movement abnormalities in schizophrenia are a well-established phenomenon that has been observed in many studies. In such studies, visual targets are usually presented in the center of the visual field, and the subject's head remains fixed. However, in every-day life, targets may also appear in the periphery. This study is among the first to investigate eye and head movements in schizophrenia by presenting targets in the periphery of the visual field.

**Methodology/Principal Findings:**

Two different visual recognition tasks, color recognition and Landolt orientation tasks, were presented at the periphery (at a visual angle of 55° from the center of the field of view). Each subject viewed 96 trials, and all eye and head movements were simultaneously recorded using video-based oculography and magnetic motion tracking of the head. Data from 14 patients with schizophrenia and 14 controls were considered. The patients had similar saccadic latencies in both tasks, whereas controls had shorter saccadic latencies in the Landolt task. Patients performed more head movements, and had increased eye-head offsets during combined eye-head shifts than controls.

**Conclusions/Significance:**

Patients with schizophrenia may not be able to adapt to the two different tasks to the same extent as controls, as seen by the former's task-specific saccadic latency pattern. This can be interpreted as a specific oculomotoric attentional dysfunction and may support the hypothesis that schizophrenia patients have difficulties determining the relevance of stimuli. Patients may also show an uneconomic over-performance of head-movements, which is possibly caused by alterations in frontal executive function that impair the inhibition of head shifts. In addition, a model was created explaining 93% of the variance of the response times as a function of eye and head amplitude, which was only observed in the controls, indicating abnormal eye-head coordination in patients with schizophrenia.

## Introduction

Schizophrenia is one of the most challenging mental disorders, with a global lifetime prevalence of 0.4% [1]–[3]. Abnormal eye movements are a well-established classifier and trait marker for schizophrenia [4]–[6]. Multiple parameters are found to be abnormal in schizophrenia, such as smooth pursuit and visual scanpaths; these are each associated with different experimental tasks reflecting various oculomotor and neurocognitive deficiencies [7]. Abnormal eye movements have been found more in familial than non-familial schizophrenia [8], and they have also been found in schizophrenic risk groups, (e.g., people with schizotypal personality [9]–[15] and first-degree relatives of patients with schizophrenia [16]–[21]). Thus, eye-movement abnormality in schizophrenia is assumed to have a genetic cause [8], [17], [22], [23] and qualifies as a robust endophenotype for schizophrenia [11], [18], [24], [25].

In such eye-movement studies, patients with schizophrenia have scanpath and visual search impairments, for example, decreased scanning length [24], [26]–[30] and fewer fixations [31]–[35]. In smooth-pursuit tasks, in which patients follow a moving target, reduced gain (ratio of eye to target velocity) and more corrective catch-up saccades were found [36]–[38]. In antisaccade tasks, in which subjects have to perform a saccade in the direction opposite that of the stimulus, larger error rates and longer latencies were associated with patients with schizophrenia than healthy controls [39]–[41]. However, antisaccade impairment does not fully meet the criteria for consideration as an endophenotype [42].

Alteration in oculomotor processing is linked to higher-order cognition. Parameters of eye movements, such as recognition performance as a function of the number of fixations in healthy controls, have been successfully linked with memory [43]–[45]. Thus, early deficits in visual processing may be related to well-known higher-order cognitive deficits in schizophrenia [46], such as memory and attentional deficits. A recent study showed that peripheral vision is impaired in patients with schizophrenia [47]. Such impairments may account for perceptual alterations and contribute to cognitive dysfunction in schizophrenia.

In summary, oculomotor impairment is a well established finding in schizophrenia research. Eye movements have been studied in a variety of experimental tasks, usually with the head fixed (for precise measurement of gaze position). However, in every-day situations, head movements are often used to perceive objects in the periphery, extending the oculomotor range to targets beyond ±10° of eccentricity in the visual field [48]. To perceive objects in the periphery, an eye-head shift is conducted, so that the object will be aligned with the fovea. In such eye-head shifts, saccadic onset usually occurs before the onset of the head movement [49], [50]. Then, prior to reaching the peripheral stimulus, the eyes begin to move in the opposite direction (the vestibulo-ocular reflex).

To our knowledge, only a single published study has investigated eye-head coordination in schizophrenia [51]; it showed that patients had longer saccade and head latencies than controls during an eye-head shift. Concerning general motor activity, dyskinesia and Parkinsonism are strongly associated with the pathogenesis of schizophrenia [52], [53]. Additionally, movement abnormalities of the face and the upper body are closely related with prodromal signs as well as psychotic symptoms (i.e., positive and negative symptoms) [54]. In this context, head movements – as a prominent part of everyday movement activity, especially during perception and social interaction – are significantly reduced during activities such as speaking [55]. Reduction in head-movement activity are observed even in first-episode patients before they receive neuroleptic medication [56]. Therefore, it is obvious that altered head movements in patients with schizophrenia may influence their eye-movement behavior.

The aim of this study is to analyze eye-head coordination in schizophrenia. In this respect, earlier research concerning only eye-movement abnormalities in patients with schizophrenia will be extended through a more naturalistic experiment for analysis of the motor base of perception. We have created a visual peripheral recognition task that promotes both eye and head movements [49]. In this task, a visual target is first presented in the center, then in the periphery. The subjects have to determine whether the two targets are equal or not. To activate the eye-head coordination system, we created two different tasks – a simple color recognition task and a more difficult Landolt-C orientation task – to investigate the hypothesis that tasks of varying complexity may induce significant differences in eye-head coordination between patients with schizophrenia and healthy controls.

## Materials and Methods

### Ethics Statement

The study was approved by the ethics committee (Kantonale Ethikkommission Bern, No. 135/09). All procedures were carefully explained to the participants, and a written study description was handed out to them. Patients were included in the study only if they had read the study description and could correctly and completely summarize all the procedures of the study. Written informed consent was obtained from all participants prior to the examination according to the tenets of the Declaration of Helsinki. All the data were recorded anonymously using random subject identifiers.

### Subjects

In total, 14 patients suffering from either schizophrenia (11 paranoid, F20.0; 1 hebephrenic, F20.1) or an acute polymorphic psychotic disorder (1 F23.0; 1 F23.1) according to ICD-10, as well as 14 healthy controls, were included in the study (the participant's demographic and clinical data are shown in Table 1). Eight of the original 22 patients were excluded (1 submitted incomplete data, 1 had glaucoma, 3 were dichromats, 2 showed nystagmus [57], and 1 had bipolar disorder). Two of the original 16 healthy controls were excluded (1 was taking antihistamines, and 1 had glaucoma). All patients with schizophrenia were inpatients at the University Hospital of Psychiatry in Bern, Switzerland. All subjects' medical histories were examined carefully. All included subjects were free of eye diseases, dichromacy, neurological diseases, diseases of the cervical spine, and shoulder/neck pain. All controls were free of neurological and psychiatric disorders and were not taking any medication. Twelve patients were being treated with atypical antipsychotics (usually risperidone or aripiprazole), one patient received both typical and atypical antipsychotics, and one patient was not taking an antipsychotic medication dose (chlorpromazine equivalent dosage [CED] in Table 1) [58]. Five patients were taking additional medication: One patient was taking antidepressants (SSRI), two were taking both benzodiazepines and antidepressants (SSRI and tetracyclic), three patients were taking mood-stabilizers (sodium valproate), and one patient was taking an opioid. The Positive and Negative Syndrome Scale (PANSS) and the Modified Rogers Scale (MRS) were used to assess overall psychopathology (Table 1).

**Table 1:** Demographic and clinical variables.

	Patients with schizophrenia	Healthy controls	Group difference1)
	(*n* = 14)	(*n* = 14)	*p*-value
Age (median [range])	30.5 (24–49)	25 (21–50)	0.022)
Gender (male/female)	4/10	7/7	0.253)
Years of education (median [range])	12.5 (9–18)	16.5 (14–21)	<0.0012)
Duration of illness (mean [SD])	12.1 (8.4)		
CED (median [IQR])	334 (138–475)		
MRS (median [IQR])	0 (0–4.5)		
PANSS positive (mean [SD])	17.0 (5.8)		
PANSS negative (mean [SD])	11.4 (4.4)		
PANSS total (mean [SD])	55.6 (15.9)		
Visual acuity (median [range])	1.00 (0.13–1.00)	1.00 (0.50–1.00)	0.162)

IQR: interquartile range.

CED: chlorpromazine equivalent dose.

MRS: Modified Roger's Scale.

PANSS: Positive and Negative Syndrome Scale.

PANSS positive: positive symptoms subscale.

PANSS negative: negative symptoms subscale.

PANSS total: total score (i.e., sum of all subscales).

1) Test of the null hypothesis that groups do not differ.

2) Wilcoxon rank-sum test, two-sided.

3)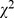 test.

### Apparatus

**Figure 1:**
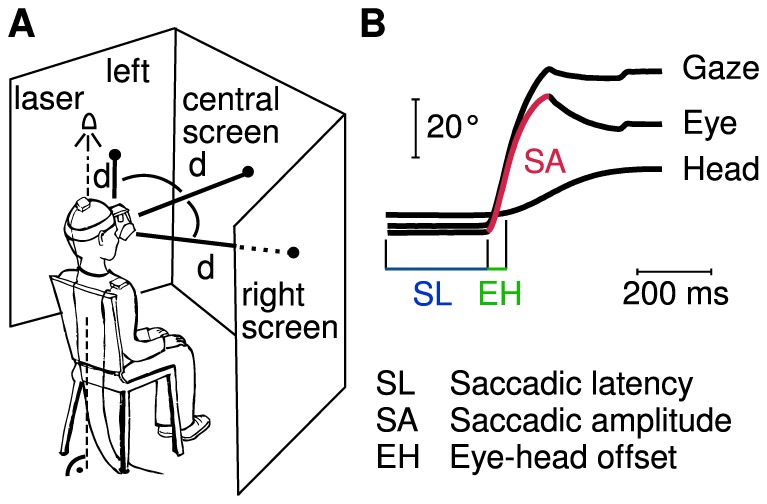
(A) Visual targets appeared at three positions (illustrated by black dots): on the left, central, and right screens. All three target positions had viewing distances of d = 80 cm. A horizontal laser was used for correct subject positioning. The peripheral targets appeared at 55° to the left and right of center. (B) A typical gaze shift with eye and head contribution. Main parameters detected were saccadic latency (SL), saccadic amplitude (SA) and eye-head offset (EH). Reprinted from www.jemr.org [49] under a CC BY license, with permission from the Journal of Eye Movement Research, original copyright 2012.

Eye movements of the dominant eye were recorded with a video-based infrared eye tracker (iView X HED-MHT, SMI, Germany) at a sampling rate of 200 Hz and a spatial resolution of 0.5–1°. Head movements were recorded using magnetic coils (Fastrack, Polhemus, USA) with a sampling rate of 40 Hz and a spatial resolution of 0.15°. A 13-point calibration within an area subtending a visual angle of 50°×15° was used. The system took into account the different centers of rotation of the eye and head. Stimuli were presented using our own software based on PsychoPy [59]. Two mirrors were used to reflect the peripheral targets to the correct positions on the left and right screens (Figure 1A). The response box had an accuracy of 1 ms.

### Visual targets

**Figure 2:**
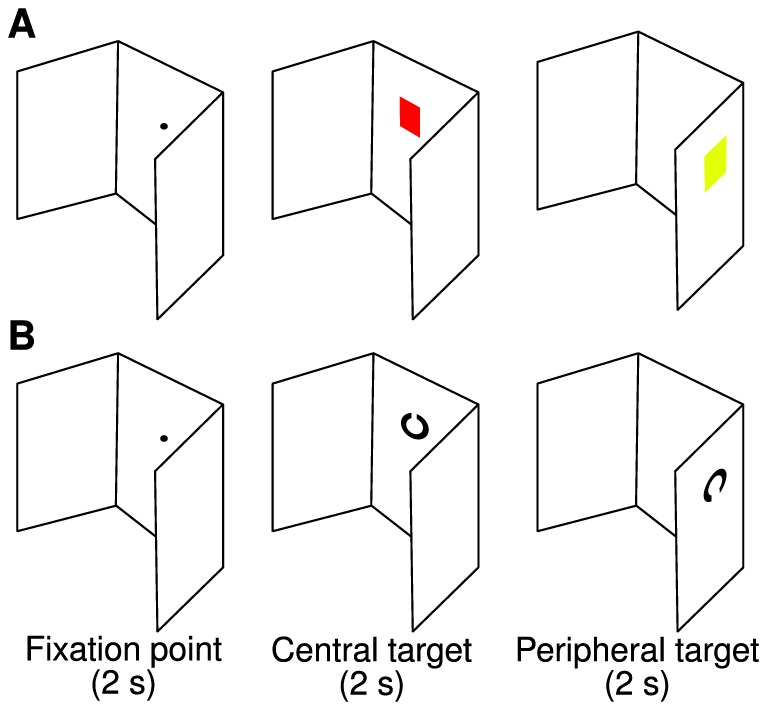
Visual peripheral recognition task with 2 exemplary trials: the color (A) and Landolt (B) tasks. Stimulus duration is stated below the figures. Reprinted from www.jemr.org [49] under a CC BY license, with permission from the Journal of Eye Movement Research, original copyright 2012.

Visual targets were presented in the center and periphery of the subject's visual field. The center of each peripheral target had an eccentricity of ±55°; the targets were projected at individual eye height (Figure 2). The visual targets were colored squares (red and yellow) or Landolt rings (upward- or downward-oriented). The targets measured 6 cm×6 cm (4.3°×4.3°) and were presented at a viewing distance of 80 cm.

### Procedure

At the beginning of the experimental session, visual acuity (Snellen chart), color vision (Ishihara test), visual dominance (Porta test), and handedness (Edinburgh inventory) were determined. All subjects were screened for eye diseases, diseases of the cervical vertebrae, neck and shoulder pain, drug abuse, and medication consumption. In the peripheral recognition task (Figure 2), a black dot – at which the subjects had to look – was presented. Then, the first target appeared in the same position, followed by a second target on either the left or right side. The task was to determine whether these two objects were identical in terms of color or orientation. The subjects were instructed to make quick and accurate responses. They pressed two buttons using their index (“Yes”) and middle (“No”) fingers of their dominant hands. The subjects were seated on a chair throughout the session and usually made no shoulder movements. However, a second receiver was attached to the right shoulder to control for small shoulder movements. A laser was used to position the subjects at the correct viewing distance. In the experimental session, each subject performed 16 training trials followed by 96 experimental trials spread across 3 blocks (32 trials per block). The trials involved color squares or Landolt rings (50% each). Likewise, peripheral targets appeared on either the left or right side (50% each). For each subject, these two conditions were randomly ordered within each block before the experiment started.

### Analysis

The data were analyzed using our own custom MATLAB software [49]. Head signals were up-sampled to 200 Hz, and the timing of those signals was synchronized to match those of the eye recordings. All eye and head data were transformed to visual angles in degrees, low-pass filtered (750°/s), and smoothed (moving average over 20 ms). Translation was performed so that the gaze and head positions were relative to the central fixation point; negative and positive angles denoted shifts to the left and right, respectively. Saccades were detected using velocity threshold algorithms: Eye-movement onset and offset were defined using 60°/s, and 15°/s as the onset and offset thresholds, respectively. Head-movement onset and offset were defined using 20°/s and 15°/s as the velocity thresholds for onset and offset, respectively. Main parameters analyzed were saccadic latency, saccadic amplitudes, eye-head offset, and number of head shifts (Figure 1B).

## Results

### Saccade latency

**Figure 3:**
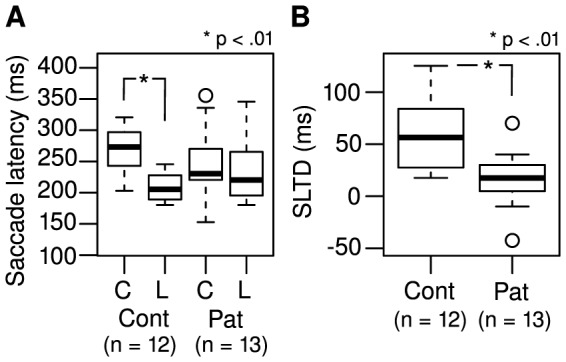
(A) Boxplots of saccade latency during the color (C) and Landolt (L) tasks. (B) Boxplots saccade latency task difference (SLTD) between controls (Cont) and patients (Pat).

A 2×2 (group × task) ANCOVA was performed to analyze saccade latency (Figure 3A) using visual acuity as a covariate. Since two controls, and one patient performed no saccades in the color task, those subjects were removed from this specific analysis. Saccade latency was generally shorter in the Landolt task than in the color task (mean difference 14.4%; F_1,23_ = 35.1; p<.001). There was no overall difference between controls and patients in terms of saccade latency (F_1,22_ = 0.007; p = .94). There was a significant interaction between group and task; that is, the saccade latency was shortest in the control group during the Landolt task (mean decreases: controls 22.3%, patients 6.5%; F_1,23_ = 12.1; p = .002). Visual acuity (F_1,22_ = 0.03; p = .88) made no significant contribution to saccade latency; further, there were no significant correlations between saccade latency and CED in the color (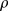 = .46; p = .11) or Landolt (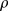 = .40; p = .17) task (Spearman's rank correlation was used because CED data were not normally distributed; Lilliefors test: D = 0.23; p = 0.048). Concerning the assumptions required for reliable ANCOVA analysis, saccade latencies were normally distributed in all four factorial groups (Lilliefors test: all ps≥.05) and had approximately equal variance (Levene's test: F = 1.54; df = 3; p = .22). Saccadic latencies in the Landolt task were subtracted from those in the color task (saccadic latency task difference, SLTD; Figure 3B). SLTD of the control group was larger than that of patients, (controls: 59.9±35.8 ms; patients: 16.2±26.7 ms; two-sided Welch two sample t-test: t(20.3)  = 3.44; p = .003). SLTD was not significantly correlated with CED (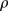 = .09, p = .78).

### Saccadic amplitude

**Figure 4:**
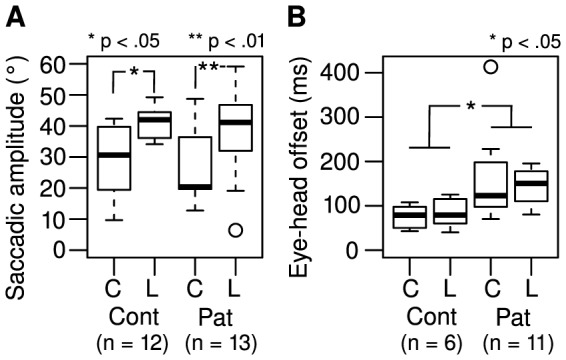
(A) Saccadic amplitude and (B) eye-head offset in the color (C) and Landolt (L) task for controls (Cont) and patients (Pat).

Median saccadic amplitudes were larger in the Landolt task than in the color task for both patients (20.8° increase) and controls (11.4° increase; Figure 4A). Saccadic amplitudes were not normally distributed in one of the subgroups (patients in the color task; Lilliefors test: D = 0.25; p = .03). Therefore, multiple statistical testing was performed, non-parametric and parametric depending on the subgroups compared. There was a significant difference in saccadic amplitudes between the color and Landolt task in both the patients (two-sided Wilcoxon signed rank test: V = 5; p = .002) and the controls (two-sided paired t-test: t(10)  = –2.93; p = .015). There was no significant difference between patients and controls in both the Landolt task (two-sided Welch to sample t-test: t(16.4)  = –0.58; p = .57) and the color task (two-sided Wilcoxon rank sum test: W = 69 p = .65).

### Head movements

Subjects did not always turn their heads during the trials. Thus, subjects were classified as head-movers and non-head movers (head shifts on fewer than 10% of trials). The proportion of head movers did not significantly differ according to group or task (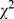 = 0.41; df = 1; p = 0.52; Table 2). The numbers of head shifts were analyzed in Table 3; this analysis revealed an increased number of head shifts in the patients during the color task (40% of trials), whereas healthy controls performed very few shifts during the color task (6% of trials; 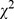 = 9.07; df = 1; p = 0.003). In the Landolt task, both controls and patients moved their heads in the majority of the trials.

**Table 2:** Head movers: Number of subjects performing more than 5 head shifts (in 48 trials) during the experiment.

	Schizophrenia patients	Healthy controls
	(n = 14)	(n = 14)
Color task	11	6
Landolt task	12	10

**Table 3:** Median number of head shifts (of total 48 trials).

	Schizophrenia patients	Healthy controls
	(n = 14)	(n = 14)
Color task	19	3
Landolt task	40.5	39.5

### Eye-head offset

Values of eye-head offset (the time between onset of the saccade and initiation of the head shift) were analyzed (Figure 4B). Only subjects who performed saccades and head shifts in both tasks were included in the analysis. Patients had greater eye-head offsets than controls did (mean increase: 89.5%; F_1,14_ = 7.1; p = .019). There was no significant main effect of task on eye-head offset (F_1,15_ = 0.45; p = .51), and there was no significant interaction between group and task (F_1,15_ = 0.72; p = .41). There was no significant covariation between eye-head offset and visual acuity (F_1,14_ = 3.41; p = .09). Concerning the assumptions required to perform an ANOVA, the data were normally distributed (Lilliefors test: ps>.05, and the subgroups had equal variance (Levene's test: F = 1.54; df = 3; p = .22). Head-offset values were not significantly correlated with CED in either of the two tasks (color: 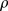 = .20, p = .56; Landolt: 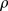 = .13, p = .70).

### Task accuracy

**Figure 5:**
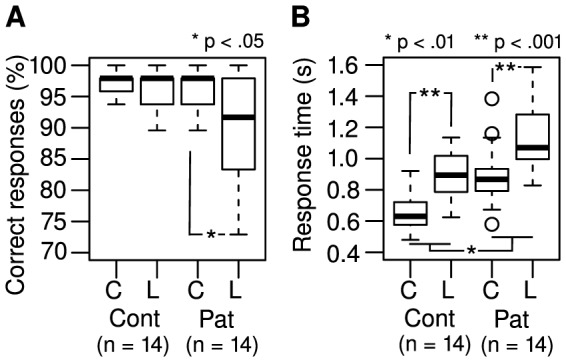
(A) Task accuracy and (B) response times in the color (C) and Landolt (L) tasks for Controls (Cont) and Patients (Pat).

The rates of correct responses (hits and correct rejections) are shown in Figure 5A. Controls had 98% correct responses in both the color task (IQR: 96%–98%) and the Landolt task (IQR: 94%–98%). Patients had 98% (IQR: 94%–98%) correct performance in the color task and 92% (IQR: 84%–97%) in the Landolt task. Correct responses were not normally distributed (Lilliefors test: D = .23, p<.001). Patients made significantly fewer correct responses in the Landolt task than the control group did (one-sided Wilcoxon rank sum test: W = 135; p = .045).

### Response times

Patients had longer response times than controls (mean increase: 30.8%; F(1,25)  = 13.1; p = .001; Figure 5B). Further, response times were increased during the Landolt task (mean increase: 31.2%; F_1,26_ = 181.9; p<.001; ). There was no significant covariation between response times and visual acuity (F_1,25_ = 1.14; p = .30), and there was no significant interaction between group and task (F_1,26_ = 0.08; p = .79). Concerning the requirements to perform an ANCOVA, response times were normally distributed (Lilliefors test: D = 0.09; p = .37), and there was equal variance among the grouping factors (Levene's test: F = 1.21; df = 3; p = .32). Response times were significantly correlated with CED in both tasks (color: 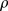 = .59, p = .025; Landolt: 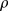 = .78, p<.001).

### Modeling subject response times

The response times of healthy controls were modeled (in the Landolt task only) by multiple (linear) regression analysis using a set of 6 predictors: saccade delay, point of regard at the time of response, saccade amplitude, saccade mean velocity, head offset, and head-movement amplitude. The best model was chosen by a stepwise algorithm containing only two predictors: reaction time as a function of eye amplitude (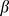 = 17.9; t = 5.36; p = .003) and head amplitude (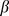 = 19.6; t = 8.41; p = .0004). The model explained a statistically significant proportion of the variance in the data (F_2,5_ = 49.9; p = .0005; R^2^ = .93). The model took 8 of 14 observations into account (since there were four non-head movers and two subjects who performed no saccades).

The resulting model was applied to the schizophrenia patients and extended by the predictors CED, PANSS positive score, PANSS negative score, and the Modified Roger's Scale (MRS). The stepwise fit of the regression model returned response time as a function of eye amplitude, head amplitude, CED, and MRS. However, this model could not explain the variance in the data well (F_4,4_ = 1.33; p = .40; R^2^ = .14). This model took 9 of 14 observations into account (since there were two non-head movers and three subjects with missing MRS).

## Discussion

The goal of this study was to investigate eye-head coordination in schizophrenia using two different visual tasks. We hypothesized that the two tasks may invoke different patterns of eye-head coordination in patients with schizophrenia and healthy controls. The results confirm the hypothesis that the characteristics of eye and head movements during a peripheral visual recognition task in patients with schizophrenia may be different from those of healthy controls.

Two different tasks were created, the Landolt-C task with two different orientations and the color task with two different colors that had do be recognized. These two task invoked a different behavioral pattern: the Landolt task caused larger saccades and longer reaction times compared to the color task indicating that subjects made fixations closer to the Landolt target and altogether required more time to solve the task. We conclude that the Landolt task triggered more foveal vision compared to the color task.

We found different patterns of saccade latencies between patients and controls (controls had long latencies in the color task but short ones in the Landolt task, whereas patients had similar latencies across both tasks). This pattern of saccadic latencies found in the control group suggests that saccadic latency may be modulated by task difficulty (i.e., the Landolt task which is associated with longer response times caused shorter saccadic latency than the color task did). On the other hand, this effect was not prominent in patients, whose saccadic latency did not differ. In our study, controls' saccadic latencies were reduced by an average of about 50 ms. Interestingly, one may speculate that patients cannot adapt to the different tasks to the same extent as controls on the basis of their pattern of task-specific saccadic latencies. This may provide a specific ocoulomotoric example of the contention advanced by other researchers that patients with schizophrenia have difficulties determining the relevance of stimuli [60]. However, further investigation is required to determine the characteristics of such a relationship. Indeed, some studies have shown that specific tasks that impose high cognitive demands (e.g., identification of objects vs. simply looking at them) can reduce saccadic latency [61]. Our data suggest that task difficulty (in our experiment, orientation detection vs. color detection) may similarly reduce saccadic latency in healthy controls, but not in patients with schizophrenia. Both controls and patients had larger saccadic amplitudes in the Landolt task; probably because subjects fixated the Landolt targets more closely at its position in the periphery and performed a larger saccade in that direction.

Patients showed an increased number of head shifts in the color task. While controls had very few head movements (3 in 48 trials on average), patients performed an average of 19 head shifts in the same number of trials. The patients used a combination of head and eye movements while performing the color task, in which normal subjects primarily use eye movements. This result is a replication of previous studies [62] and can be observed in other types of tasks, for example during silent reading [63]. Similarly, patients with schizophrenia performed more head movements than healthy controls in a silent reading study. It appears that patients may show uneconomic over-performance in tasks that do not require head movements; this is possibly caused by alterations in frontal executive functions that impair the inhibition of head shifts. Likewise, failure of inhibitory mechanisms has also been discovered in antisaccade tasks [64]; such failure was interpreted as an inhibition deficit in the executive control of action [65].

The question why humans exhibit differing propensity to move the head in gaze shifts has been addressed by a few studies. Head-mover/non-head mover variation depends on ocular motor behavior; head movers tend to maintain the eyes within a narrower ocular-motor range compared to non-head movers [66]. Manipulating head movements (using a collar) caused reduced head and increased eye amplitudes [67]. Our study adds that the interplay of the head and the eye amplitude are the main components explaining reaction time during the tasks. This model takes into account the different tendencies to move the head among subjects.

Patients gave significantly fewer correct responses than controls in the Landolt task, whereas in the color task, all subjects had overall good performance. It seems that increasing task difficulty generates different results in controls and patients, possibly because attentional ability might become more relevant with increasing task difficulty. Further, patients differed from controls in terms of timing characteristics: patients had longer eye-head offsets and response times than controls in both tasks, supporting the findings of a previous study of eye-head coordination in patients with schizophrenia [51]. This result might be due to the patients' cognitive impairments, medication consumption, or both. It is well-known that eye-movement parameters are affected by certain drugs, especially those that affect the central nervous system. Benzodiazepines and second-generation antipsychotics can alter eye movements (e.g., generating a decrease in saccadic peak velocity [68], [69]), but they might also affect other eye-movement parameters. Therefore, we have expressed our results regarding medication in terms of CED. We could not find any significant correlations between medication on the one hand and eye or head parameters on the other. Even though medication dosage does not seem to be associated with the eye and head parameters observed in this study, medication effects cannot be completely excluded. We found that response time was significantly correlated with CED.

A general issue in correlation analysis concerns the fact that only two variables are taken into account. This two-dimensional view may often fail to capture the complex interactions between behavioral measures, pathology, and medications. Therefore, we have modeled response time using a set of six predictors (which consists of eye and head-movement characteristics). Among a subset of 8 of the 14 healthy controls (those subjects who performed both eye and head movements), our model explained 93% of the variance in response time. The 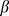 values of the model suggest that each additional degree of eye and head shift corresponds with increases in response time of about 18 and 20 ms, respectively. We interpret these results to mean that response time is largely explained by both eye and head shifts. Interestingly, we could not identify a similar simple model in the patients with schizophrenia, in whom we also accounted for medication dosage and psycho-pathological scales as potential predictors. The failure to find a simple equivalent model for patients may be due to their impairments in sensorimotor integration, which cause alterations in their eye-head coordination patterns.

We now address some limitations of this study. First, most patients were taking medication, which may affect their oculomotor functions. Second, we cannot conclude that these results are specific to schizophrenia, since no other pathological groups were investigated (e.g., patients with mood disorders). However, a recent review [70] analyzing studies published in 1986–2011 concluded that findings concerning anxiety and mood disorders have failed to capture trends in oculomotor impairment.

In conclusion, this study provides new evidence that patients with schizophrenia may exhibit different eye and head movement behavior during peripheral visual recognition tasks from healthy controls. We hope that these results will stimulate further investigation of oculomotor impairments in more naturalistic settings in which both eye and head shifts are demanded. Future studies should apply similar techniques to related disorders; longitudinal studies should also be performed to draw associations between psychopathology and prognosis on the one hand and altered eye-head movements on the other, likewise as it has already been explored in eye-movements studies [71], [72].
